# Beacon Success Rate versus Gateway Density in Sub-GHz Sensor Networks

**DOI:** 10.3390/s23239530

**Published:** 2023-11-30

**Authors:** Başak Can, Bora Karaoğlu, Srikar Potta, Franklin Zhang, Artur Balanuta, Muhammed Faruk Gencel, Uttam Bhat, Johnny Huang, Pooja Patankar, Shruti Makharia, Radhakrishnan Suryanarayanan, Arvind Kandhalu, Vinay Sagar Krishnamurthy Vijaya Shankar

**Affiliations:** Amazon Lab126, 1100 Enterprise Way, Sunnyvale, CA 94089, USAjohnnhua@amazon.com (J.H.);

**Keywords:** beacon collision, beacon interval, Carrier-to-Interference ratio, frequency hopping, gateway, GFSK, Internet of Things (IoT), IEEE 802.15.4, latency, Wide Area Network (WAN), sensors

## Abstract

Multiple Gateways (GWs) provide network connectivity to Internet of Things (IoT) sensors in a Wide Area Network (WAN). The End Nodes (ENs) can connect to any GW by discovering and acquiring its periodic beacons. This provides GW diversity, improving coverage area. However, simultaneous periodic beacon transmissions among nearby GWs lead to interference and collisions. In this study, the impact of such intra-network interference is analyzed to determine the maximum number of GWs that can coexist. The paper presents a new collision model that considers the combined effects of the Medium Access Control (MAC) and Physical (PHY) layers. The model takes into account the partial overlap durations and relative power of all colliding events. It also illustrates the relationship between the collisions and the resulting packet loss rates. A performance evaluation is presented using a combination of analytical and simulation methods, with the former validating the simulation results. The system models are developed from experimental data obtained from field measurements. Numerical results are provided with Gaussian Frequency Shift Keying (GFSK) modulation. This paper provides guidance on selecting GFSK modulation parameters for low bit-rate and narrow-bandwidth IoT applications. The analysis and simulation results show that larger beacon intervals and frequency hopping help in reducing beacon loss rates, at the cost of larger beacon acquisition latency. On the flip side, the gateway discovery latency reduces with increasing GW density, thanks to an abundance of beacons.

## 1. Introduction

This paper presents an analysis of the system capacity of a Wide Area Network (WAN). The WAN is served by a set of Gateways (GWs). Each GW serves multiple End Nodes (ENs). The WAN can serve various environments, including houses, apartment complexes, farms, or enterprises with smart sensor systems. The WAN is an Internet-of-Things (IoT) network that is connected to a cloud-based application. These applications may include functionalities such as facilitating centralized control, the monitoring of smart home devices, or offering real-time tracking for the location and activities of pets, for instance. The GWs can be any device with a connection to the Internet and the cloud server, facilitating the interconnection of sensor data with the application. These devices are equipped with multiple types of connectivity hardware (HW). First, they have a narrow-band IoT capable communications HW that can directly connect to the sensors. Secondly, they have dedicated Wi-Fi connectivity HW to route the sensor data to the Internet/cloud server. Typically having access to a power source, the GWs become more intricate to simplify the protocol for the sensors. The number of sensor devices and GWs in each home, apartment complex, or enterprise is expected to grow exponentially. Hence, it is important to understand the limits of such networks in terms of the total number of devices that can be supported with good Quality of Service (QoS). These devices are installed by the general public in locations in and around houses or neighborhoods. Given the use of sub-GHz carrier frequencies within the Industrial, Scientific, and Medical (ISM) bands, these WAN networks will experience interference from other networks, such as Sigfox, LoRa-WAN, and Zigbee, etc. This study presents an in-depth model for evaluating the performance of such networks in relation to the size of coexisting devices. The performance evaluation is provided for the coverage area, GW density, latency, and Packet Success Rates (PSR).

**Figure 1:**
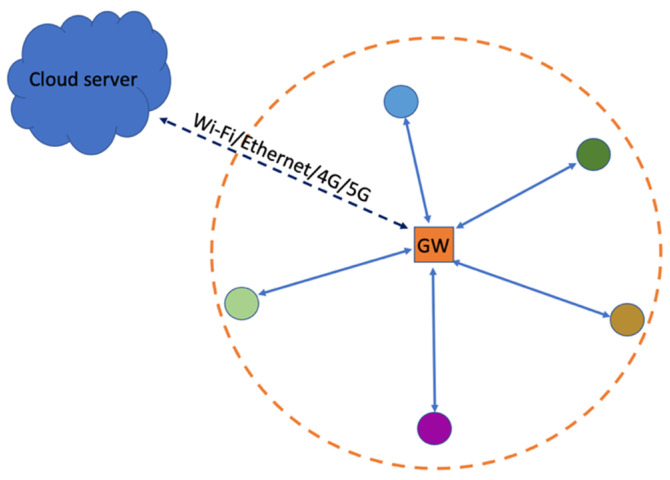
Star network topology for each Gateway, facilitating the discovery and connection of multiple sensors.

**Figure 2:**
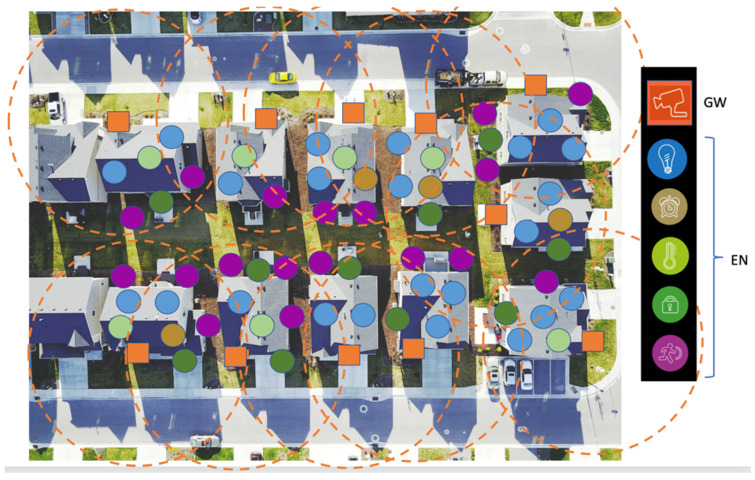
Multiple GWs co-existing in a (sub)-urban neighborhood with numerous sensors.

GWs have access to a power outlet and the Internet; hence, they can be used as central devices with more sophisticated system operations to serve and simplify the WAN sensors. In this paper, a star network topology is assumed, as depicted in Figure 1, where IoT sensors are represented by different colored circles connecting to the GW [1]. Having a multi-hop mesh topology is not recommended due to the already low bit-rates available in each link. Coverage issues are overcome by GW diversity instead [2]. With GW diversity provided by the network operator, sensors can access cloud services via the closest neighbor’s GW or via the user’s own GW. ENs serve as sensors for various IoT applications and are typically battery operated. These applications can include motion sensing, smart lock and contact sensing, geo-fencing, smart metering, temperature sensing, asset/pet tracking, and any other IoT applications, as illustrated in Figure 2. All GWs can connect to the same cloud server, where the reach of each GW can be extended to the scale of a WAN. Since the WAN operates in an unlicensed frequency spectrum, it needs to coexist among other WANs, as illustrated in Figure 2. Each of the star networks, depicted by an orange circle centered on its GW, functions autonomously without any dependence on other star networks. Each GW can access to the same cloud server. For such a network, further system optimizations among the GWs can be performed centrally via the cloud server. With narrow-band connectivity hardware suitable for low-bitrate IoT applications, the coverage area of each GW can span to few hundred meters, causing interference to the neighboring GWs. Hence, it is important to understand the system performance within such an interference prone environment.

In this study, system capacity is defined in terms of the number of Gateways (GWs) needed to serve a given geographical area. The analysis is provided for PSR and latency versus the number of ENs as they join the network. The analysis is extended to include the neighboring GWs that interfere with the primary GW. The paper presents how the distance to a GW is mapped to a PSR and Co-Channel Carrier-to-Interference (C/I) ratio. Channel access is assumed to be over the sub-GHz ISM band, also known as the 915 MHz band in the U.S. and 868 MHz band in Europe. Hence, the system is impacted not only by intra-network interference from neighboring GWs, but also by inter-network interference from other networks. While interference in the environment cannot be controlled, intra-network interference can be reduced by using efficient channel access algorithms such as Listen Before Talk (LBT) and frequency hopping, or Time Slotted Channel Hopping (TSCH) [1,3]. To control the collisions in random medium access, various techniques such as carrier sense and random backoff mechanisms can be used [1,2,3,4,5,6,7,8,9,10,11]. Collisions refer to the simultaneous use of the same radio channel by two or more devices transmitting at the same time. Collisions cause co-channel interference, occurring when there are one or more interfering signals present in the channel during communication between the desired transmitter and receiver. The LBT requires the transmitter to transmit only when the channel is determined to be idle by Carrier Activity Detection (CAD). If the CAD determines the medium to be busy, the backoff mechanism requires each EN to wait for a random period before attempting a retransmission. This improves the performance by reducing collisions. However, the LBT scheme suffers from hidden-node problem, where two nodes might be out of coverage with each other while causing interference to a given GW [12].

The LBT mechanism is used for Uplink (UL) transmissions by the ENs to avoid interfering with each other and with the other beacons of neighboring GWs. This improves the performance significantly [1,2,3,4,5,6,7,8,9,10,11]. Nonetheless, in the Downlink (DL), GWs do not employ the LBT mechanism. Instead, GWs are required to periodically transmit their high-priority beacons, sending one beacon at each beacon interval. Ensuring this procedure is essential to prevent ENs from losing synchronization with the desired GW, which could lead to network disconnection, particularly when consecutive beacon signal losses occur. This study primarily examines the performance of beacon collisions between multiple GWs and their success rates as received by ENs.

### 1.1. Introduction to IEEE 802.15.4 Specification

In this section, a brief introduction to Institute of Electrical and Electronics Engineers (IEEE)-802.15.4 standard is provided.

The IEEE-802.15.4 standard provides specifications for the physical layer (PHY) and medium access control (MAC) sublayers, specifically tailored for wireless connectivity at low data rates involving fixed, portable, and mobile devices with either no battery or stringent battery consumption constraints. Numerous investigations explore the connectivity performance of diverse PHYs delineated in the IEEE802.15.4g (2012) amendment to the IEEE802.15.4 (2003) standard. This amendment introduces 31 PHYs specifically designed for Smart-metering Utility Network (SUN) applications, grouped into three modulation families: Frequency Shift Keying (FSK), Offset Quadrature Phase-Shift Keying (O-QPSK), and Orthogonal Frequency Division Multiplexing (OFDM) [13]. With data rates ranging from 25 kbps to 800 kbps, these PHYs function across two frequency bands: 2.4 GHz and sub-GHz [1].

The various PHY specifications outlined in the standard accommodate devices operating in diverse geographic regions [1]. In summary, the specification is designed for short-, medium-, and long-range communication with low data rates, low power consumption, and low complexity. In this paper, Gaussian Frequency Shift Keying (GFSK) modulation with a data rate of 50 kbps is assumed for 802.15.4-based networks, particularly for Narrowband IoT (NB-IoT) applications. This choice is motivated by various factors presented, as follows.

- Low Power Consumption: GFSK modulation is known for its energy efficiency, making it suitable for low-power devices and battery-operated sensors. In NB-IoT applications, where devices may need to operate on battery power for extended periods, minimizing energy consumption is crucial.
- Spectral Efficiency: GFSK modulation ensures the effective use of the frequency spectrum by allowing data transmission in a comparatively narrow bandwidth. This efficiency is achieved via the integrated Gaussian Pulse Shaping filter. This becomes particularly significant in situations where the available frequency spectrum is constrained, a common occurrence in IoT deployments. Moreover, GFSK modulation, being a constant envelope modulation, avoids issues of spectral regrowth caused by Power Amplifier Nonlinearities.
- Moderate-Range Communication: The GFSK modulation with a narrow bandwidth is designed for moderate-range communication, typically within a few hundred meters. This makes it suitable for applications where devices are located in close proximity to a building, such as home automation, industrial monitoring, or healthcare settings.
- Cost-Effective Implementation: GFSK modulation’s simplicity facilitates cost-effective radio transceivers, making it an appealing choice in cost-sensitive IoT deployments.
- Compatibility with the IEEE-802.15.4 Standard: GFSK modulation with a data rate of 50 kbps aligns with the specifications of the 802.15.4 standard. This ensures interoperability between devices from different manufacturers that adhere to the same standard, fostering a more open and scalable IoT ecosystem.
- Suitability for Low-Data-Rate Applications: The 50 kbps data rate is well-suited for many IoT applications where the focus is on transmitting small amounts of data intermittently. Examples include sensor data, control signals, and sensor status updates.

In summary, the choice of 802.15.4-based GFSK modulation with a 50 kbps data rate in NB-IoT networks is driven by the need for low power consumption, spectral efficiency, and suitability for medium-range, low-data-rate communication in cost-sensitive IoT applications.

### 1.2. Related Work

The contributions of this paper along with related work and a literature review are summarized as follows. In [14], the authors introduce the Listen Interval Adaptive Adjustment (LIAA) scheme to enhance the energy efficiency and reduce the end-to-end delays in event-sparse IoT systems, focusing on wireless sensor networks. LIAA includes three strategies (BALI, CL, and RALI) leveraging energy imbalances to optimize listen intervals, resulting in a 24.03%, 23.45%, and 39.41% reductions in delays for each strategy. The paper also addresses challenges in energy consumption and security. The paper in [15] addresses challenges in TSCH networks in industrial environments, proposing a distributed PHY and parent selection heuristic for slot bonding multi-PHY TSCH sensor networks. The paper in [16] introduces g6TiSCH (IPv6 over the TSCH mode of IEEE 802.15.4e). g6TiSCH enables nodes with multiple radios to dynamically switch between them based on link quality, creating a trade-off between latency and power consumption. An experimental evaluation on a 36-node testbed demonstrates that g6TiSCH outperforms traditional 6TiSCH stacks on individual physical layers, achieving a lower latency and network formation time while maintaining comparable battery lifetime. The paper in [17] explores how the Industrial Internet of Things (IIoT), which uses wireless connectivity and IP networking, can revolutionize industries such as Cyber-Physical Systems (CPS). Examples of CPS include industrial control systems, water systems, robotics systems, and smart grids, etc. To meet industrial demands, the paper focuses on the TSCH MAC and surveys 76 different schedulers, organizing them into categories such as Centralized, Collaborative, Autonomous, Hybrid, and Static. The analysis shows a trend of increased complexity alongside the adoption of centralized principles. The paper also outlines prospective research directions, including fault tolerance concerning latency and reliability, as well as scalability. The paper in [18] addresses challenges in beacon scheduling and Guaranteed Time Slot (GTS) allocation within IEEE 802.15.4 Deterministic and Synchronous Multichannel Extension (DSME) networks, commonly used for IoT. It proposes a non-conflicting beacon scheduling mechanism using association order to tackle slot collisions and introduces a distributed multi-channel DSME-GTS schedule for optimal allocation across channels. The goal is to minimize time slot usage while maximizing channel utilization. The simulation results in the paper show that the proposed mechanisms outperform existing schemes in energy efficiency, transmission overhead, scheduling efficiency, throughput, and latency.

### 1.3. Contributions of the Paper

This section provides the motivation and introduction to the contributions of this paper. The previously mentioned works explore various aspects of improving MAC efficiency within IoT systems. In adaptive schemes, difficulties emerge in link adaptation via dynamically changing Modulation and Coding Scheme (MCS) or in formulating scheduling decisions based on link quality. Such difficulties are especially relevant in scenarios where Channel State Information (CSI) might not be readily accessible. This constraint is particularly notable in low-bitrate IoT systems characterized by extended airtimes, heightened protocol latencies, and operation in unlicensed bands, making the Channel State Information (CSI) obsolete by the time it is available for decision making. To address these challenges, this study takes a different approach by focusing on fixed bit-rate strategy and employing simple periodic beaconing alongside TSCH. The aim is to simplify the protocol and to reduce the overhead, especially for low-bitrate End Nodes (ENs). While this simplification mitigates certain complexities, it introduces challenges related to intra-network interference and its impact on latency, which are thoroughly analyzed in this paper.

The findings in this study indicate that enhancing beacon success rates can be achieved by implementing larger beacon intervals and increasing the number of channels. Introducing GW diversity proves advantageous in reducing beacon acquisition latency and expanding the coverage area [2]. In urban settings, the abundance of GWs enhances the potential for Gateway diversity. Despite a potential increase in DL beacon collision rates, the significant benefits of spatial diversity become apparent. Each GW plays a role in subdividing the path-loss into smaller segments, thereby improving the overall network performance. Furthermore, the strategic use of larger beacon intervals proves advantageous in augmenting network capacity by reducing beacon collision rates. This emphasizes the significance of strategically utilizing GW diversity and fine-tuning beacon intervals to optimize network performance in urban environments. Additionally, this paper introduces a co-channel interference model that dynamically considers partial overlaps from other co-channel interferers. This model takes into account the relative power of these interferers compared to the desired signal, providing a comprehensive understanding of interference dynamics in the network. As a result, it offers insights into the fine-tuning of various system parameters. The model is used to derive the relationship between beacon collisions and packet loss rates. The paper presents guidance on the selection of GFSK modulation parameters for low bit-rate and narrow-bandwidth IoT applications.

This paper is organized as follows. In Section 2, the system model regarding the channel access scheme, interference, and path-loss models is presented. Section 3 presents the PHY layer model with GFSK modulation parameters specifically tailored for narrowband IoT sensors. Such parameters have not been previously explored, making it of significant importance for narrowband devices. The theoretical analysis of the beacon collision probability versus network size is presented in Section 4. The simulator architecture is presented in Section 5. Finally, the numerical simulation results of the collision probability versus network density are presented in Section 6 with the PHY and Medium Access Control (MAC) layer models. The conclusions from this study are presented in Section 7.

## 2. System Model

In this section, the channel access and interference model, along with the path loss model, are presented, all of which are used in our simulations. The IEEE-802.15.4 standard specifies the MAC and PHY layers of Low-Rate Wireless Personal and Wide Area Networks [1]. It provides the basis of various IoT standards that find applications in smart home automation and sensor networks. Hence, the system parameters selected in this study are based on the 802.15.4 specification.

(A) **Channel Access**

In this section, the channel access scheme is presented. Each GW periodically broadcasts beacons that allow the ENs to discover the GW and join its WAN. In other words, the ENs connect to a nearby GW by scanning its advertising beacons. The Beacon frame format is used for broadcasting radio configuration parameters by the GWs. Endpoints use the information in the Beacon frame to choose a Gateway and for maintenance of the Gateway–Endpoint connection, including power control and channel hopping.

ENs working in synchronous connection mode should be synchronized with a GW before an uplink or downlink transmission is performed. The synchronization mechanism works by searching for a periodic frame sent by GWs, namely Beacon frames. The time between the two consecutive beacons is referred to as the beacon interval or beacon period. When initially joining the WAN network, the ENs detect available GWs by performing a beacon scan process where all channels are swept for beacon preambles. When a preamble is detected on a channel, the EN will lock onto the channel in an attempt to receive a beacon. If a beacon is successfully received and decoded, the EN will use the timing and parameters transmitted within the payload of the beacon to acquire synchronization with that particular GW.

Frequency Hopping Spread Spectrum (FHSS) is used and the hopping sequence is determined by the GWs. The beacon’s payload provides synchronization information such as GW seed and beacon sequence number information. This is used by the EN for frequency hopping sequence calculations. Time synchronization is maintained by periodic beacon transmissions by each GW. The beacon payload can also carry additional information about the state of the GW, such as the current GW load, cloud connectivity status, roaming support, or information about the other nearby GWs belonging to the same network. These additional data can influence GW selection by the EN when multiple GWs are available. Beacons provide time and channel synchronization and network discovery across the network. The periodicity of the beacons provides time synchronization, while the sequence number and seed provide channel synchronization. Each WAN is served by one GW, but the EN can choose from multiple GWs available in the NW. After beacon discovery, if an EN can no longer receive consecutive beacons, for, e.g., at least three times, then it falls out of the WAN and needs to re-scan for beacons from other GWs.

The WAN network is a synchronized system following a star network topology, where there are specific time slots for transmission and reception. There are fixed 160 time slots from the start of beacon transmission to the start of consecutive beacon transmission. Slots 0–3 are reserved for beacon transmission, while the remaining 156 time slots are available for data transmission shared across the entire WAN. The beacon interval and the number of slots can be programmed to different values to optimize the overall network performance in terms of collision probability and the number of devices that can be supported with a good QoS.

FHSS uses 69 channels in the ISM band, starting from a 902.2 MHz carrier frequency and up to 927.8 MHz with 2 MHz spacing. The channel calculations for the beacon broadcasting and Endpoint uplink or downlink transmissions are calculated separately. The result of the calculations is a hopping sequence that is derived from the total number of channels available for upcoming transmissions. The hopping sequence is calculated by shuffling the channel list through a channel shuffler. This channel shuffler is shuffled by iterative calls to a Pseudo Random Number Generator (PRNG) seeded by the seed and sequence number. This PRNG is of the Linear Feedback Shift Register (LFSR) type. This shuffle provides a hopping sequence that guarantees all channels will be visited exactly once within each full hopping cycle. The channel shuffler used for beacon calculation for DL is different from those used for UL data transmission due to differences in periodicity. Each beacon is sent in one channel. Hence, the beacon channel shuffler is only drawn once every beacon interval. The data transmissions in UL can be performed in one out of 156 slots. Hence, the data channel shuffler is drawn 156 times within each beacon. This way, the GW can also determine which channels to listen to during UL.

**Figure 3:**
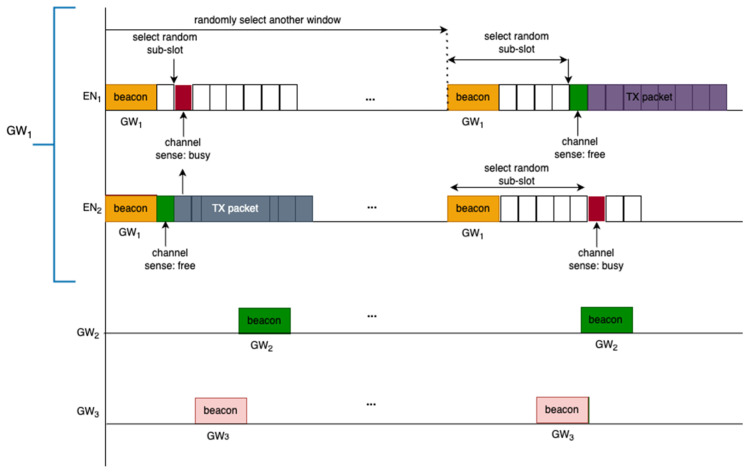
The channel access mechanism employing LBT.

The previous paragraphs described how a synchronized connection is established between an EN and a GW based on the beacons. As part of the mechanism to minimize interference, non-beacon packets (i.e., the data packets) use the LBT mechanism. When an EN has a packet that needs to be transmitted in the queue, it first randomly selects one of the next four transmission slots relative to the beacon start time. It then selects one out of the six LBT sub-slots by drawing from a geometric random distribution. It will then schedule the LBT operation to occur at the correct time based off of the time slot and LBT sub-slot. When the scheduled LBT operation occurs, the EN first listens to determine if anyone else is actively transmitting a packet by entering in preamble detection mode. If the medium is available, i.e., when there is no signal detected from other devices, then the EN starts its UL data transmission. If the medium is detected to be busy, then the EN backs off for a random duration by selecting one of the next sets of four transmission slots. From there, it will select an LBT sub-slot where it will repeat the LBT process. This is illustrated in Figure 3. The connection to a GW is established via the successful discovery of the beacon in the DL and when the GW registers the EN by receiving the UL packets from it. This channel access scheme reduces the chances of packet collisions within the WAN as ENs join the network, but it does not guarantee a collision-free medium. An analysis of such capacity will be presented in Section 6. The analysis shows that the system capacity depends on the number of channels available for FHSS, the number of ENs in the desired WAN, the number of ENs in the neighboring WAN, the number of GWs in the area, and the airtimes of the packets (beacon interval, beacon duration, UL packet durations, and UL packet rates).

(B) **The Co-channel Interference Model**

In this section, the co-channel interference model is presented. The packets can potentially collide due to the shared medium access with a finite number of channels and finite number of transmission slots. Collisions occur when transmission attempts occur on the same channel and overlap in time. Within the same WAN network, this occurs when the same transmission slot and same LBT sub-slot are selected. When both of these conditions are met, the same channel is selected, and LBT returns channel clear during LBT for both transmissions, leading to both devices starting TX. Although collision avoidance is in place for the ENs within the coverage of a given GW, collisions on a beacon can still happen from neighboring devices. This is illustrated in Figure 3, where the beacon of GW_2_ is interfered by the UL packet from EN_2_ which is connected to GW_1_.

**Figure 4:**
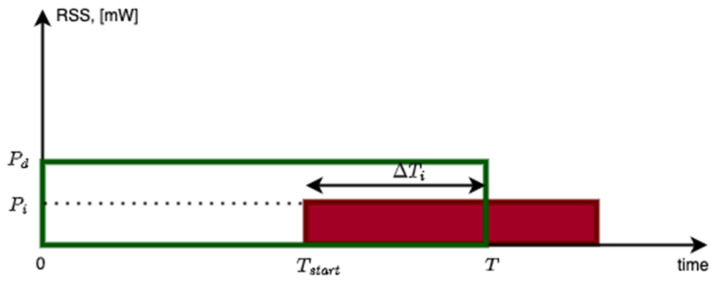
Occurrence of co-channel packet collision within a data packet.

Co-channel collisions can happen over a portion of the desired packet. Let $\Delta T_{i}$ represent this portion of time and let T represent the duration of the desired packet. Let $r_{i}≝\frac{\Delta T_{i}}{T}$ represent the interferer overlap ratio for the interferer i. Let $P_{d}\;\mathrm{and}\;P_{i}$ represent the Received Signal Strength (RSS) in mW from the desired transmitter and the interferer i, respectively. These definitions are illustrated in Figure 4. In this illustration, the time boundaries of the intended packet are denoted by a green rectangle, and the time boundaries of the interfering packet are indicated by a red rectangle. The y-axis corresponds to the RSS of each packet upon arrival at the receiver. Let $\frac{C}{I}$ represent the carrier to interference ratio (in dB) when a co-channel interferer is present. With these definitions, the carrier to interference ratio can be derived as:

(1) $$\frac{C}{I}=10\times log_{10}\{\frac{P_{d}}{\Sigma_{i}P_{i}\times \frac{\Delta T_{i}}{T}}\}=10\times log_{10}\{\frac{P_{d}}{\Sigma_{i}P_{i}\times r_{i}}\}\;\left[\mathrm{dB}\right]$$

In this formula, the overlapping interference power from all interferers is aggregated to determine the overall carrier-to-interference ratio.

The PSR is the probability at which packets can be received successfully. The PSR can therefore be related to the Packet Error Rate (PER) by the following formula:

(2) PSR≡1−PER

The PSR depends on various factors such as:

1. The distance, *d*, between the receiver and the transmitter, which determines the RSS at the receiver,
2. The receiver sensitivity for the selected MCS,
3. The carrier to interference ratio (C/I) and number of interfering events.

**Figure 5:**
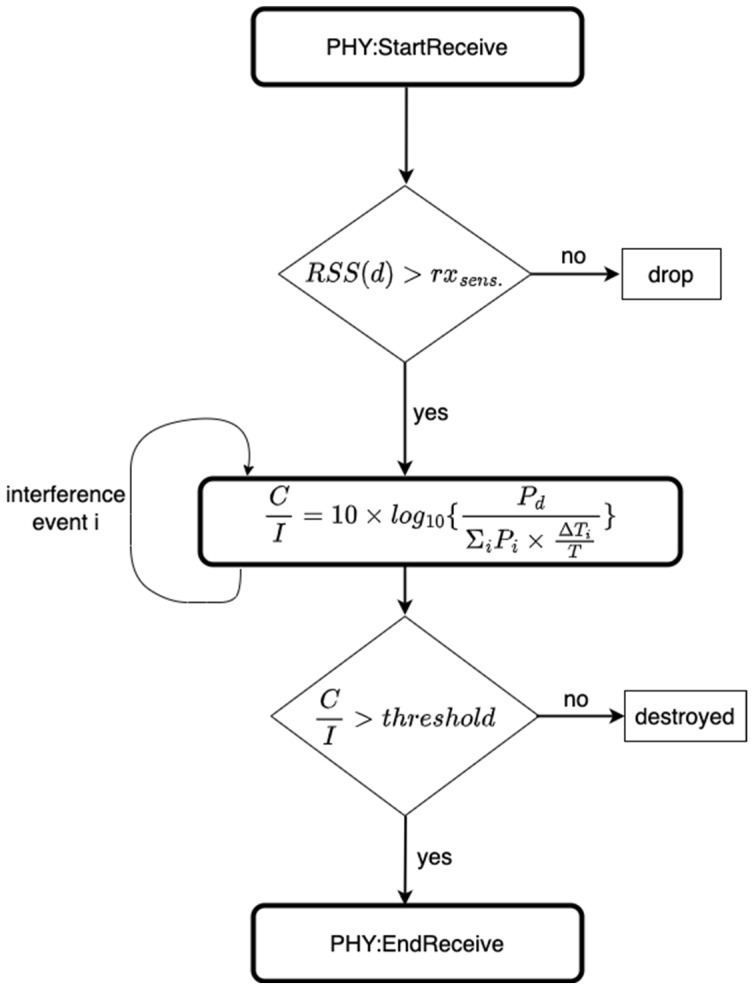
The model encompassing path-loss, receiver sensitivity, and co-channel interference.

The collision and interference model in the simulator considers all these parameters before a packet can be marked as successfully received or dropped. A packet drop can be caused by unfavorable conditions such as large interference or path-loss. This is illustrated in Figure 5. The term $rx_{sens.}$ represents the receiver sensitivity in dBm. For the packets to be considered as successfully received, the RSS of the desired signal at distance d from the transmitter needs to be greater than the receiver sensitivity, i.e., $RSS\left(d\right)>rx_{sens.}$ Furthermore, once this condition is satisfied, the C/I needs to be above a threshold in order for the packet to be received successfully. This threshold depends on the selected MCS in the communication link. Let $\gamma_{th}$ represent this threshold in dB. The higher-bitrate MCSs require a larger threshold and larger sensitivity. These thresholds will be presented in detail in Section 3, assuming that GFSK modulation is used for the MCS.

If there are two packets colliding in time, and one of them is from the desired signal link, and the other one is from the interfering link, then the PSR can be related to the Bit Error Rate (BER) as follows:

(3) $$PSR(C/I)\;=\;{\left(1-r\;\times BER\left(C/I\right)\right)}^{\left(\left(r\times p\;\right)\right)}$$

where the term p represents the number of bits in the packet, and the term r, 0≤r≤1, represents the overlap ratio of these two packets/links over the course of the desired packet (ref. Figure 4). The BER is applicable to the portion of the packet under interference. This is derived from the fact that all the bits under interference need to be correctly received in order for the packet to be successfully received [19]. This PSR derivation is made for an uncoded wireless link, which is assumed in the PHY layer. If error-correcting coding is used, its coding gain can be introduced to the C/I value based on measurements. In this derivation, it is assumed that the only impairment in the link is interference as captured in the C/I value. Packets with a larger number of payload bits require a stronger C/I value in order to achieve the same PSR. The PSR depends on the number of payload bits under interference. Smaller collision time overlaps, represented by the ratio r, result in favorable conditions for surviving co-channel interference. The worst case is when r=1 which represents the case of a full overlap across the desired packet. In such a case, the entire packet will be under interference. The numerical results with this model will be presented in Section 6.

(C) **Path loss Model**

In this section, the path loss model is presented for the US 902–928 MHz ISM band in an outdoor urban area. The path-loss (in dB) is defined as the total propagation loss between the transmitter and the receiver and can be defined as:$\;PL\left(d,f\right)\equiv TxP-RSS\left(d\right),\left[dB\right]$, where the term $RSS\left(d\right)$ in dBm represents the received signal strength at a distance, d [m]. The term TxP [dBm] represents the transmitted power at the transmitter. The term Received Signal Strength Indicator (RSSI) [dBm] represents the estimate of RSS, as reported by the receiver. Additional insertion losses in [dB] due to analog RF hardware losses can be added to this formula, depending on where the $RSS\left(d\right)$ refers to. This is relevant if the *RSS* is reported for the antenna port of the receiver, whereas the signal is processed at the chipset port. The path loss depends on various factors such as the channel frequency, distance between the transmitter and receiver, and random shadow fading variations caused by the environment [20,21,22,23,24,25,26,27,28,29]. In most of the models published to date, the path loss can be modeled as follows:

(4) $$PL\left(d,f\right)=PL_{0}\left(d_{0},f_{0}\right)+10\times n\times log_{10}\left(\frac{d}{d_{0}}\right)+20log10\left(\frac{f}{f_{0}}\right)+X\left(0,\sigma \right),\;[\mathrm{dB}].$$

where the term ${PL}_{0}(d_{0},f_{0})$ represents the path loss at reference distance $d_{0}$, and on reference channel frequency,$\;f_{0}$. The term n represents the path loss exponent, the term d is the distance between the GW and the EN, and f is the channel frequency in Hz. The term $X\left(0,\sigma \right)$ (in dB) is a random variable that represents the large-scale shadow fading effects, where its mean is zero, and its standard deviation is σ, expressed in decibels. The field measurements show that it has a log-normal distribution.

The carrier frequency of the radio waves affects the overall path-loss. Typically, higher frequencies exhibit higher path-loss compared to lower frequencies. This is attributed to smaller wavelengths associated with higher carrier frequencies, making the signal more susceptible to diffraction and scattering, thereby reducing the signal strength.

The path-loss exponent, n, can vary based on terrain type and the presence of non-uniform buildings. In practical scenarios, the path-loss exponent is not consistent across a wide area, as assumed in Equation (4). Its variation can be significant and is influenced by the surrounding environment and its dimensions. Several factors contribute to this variability. Firstly, the path-loss exponent tends to be higher in urban areas compared to rural areas. Urban environments, with more buildings and obstacles, lead to increased scattering and absorption of radio waves. Additionally, the presence of non-uniform buildings plays a role in influencing the path-loss exponent. For instance, an area with numerous tall buildings will have a higher path-loss exponent than an area with mostly shorter buildings. This is because tall buildings can obstruct radio waves, resulting in a decreased signal strength. The path-loss exponent serves as a crucial factor for determining the range of a wireless communication system. If the path-loss exponent is excessively high, the signal strength might be below the sensitivity of the receiver. This can lead to dropped connections or poor PSR.

**Figure 6:**
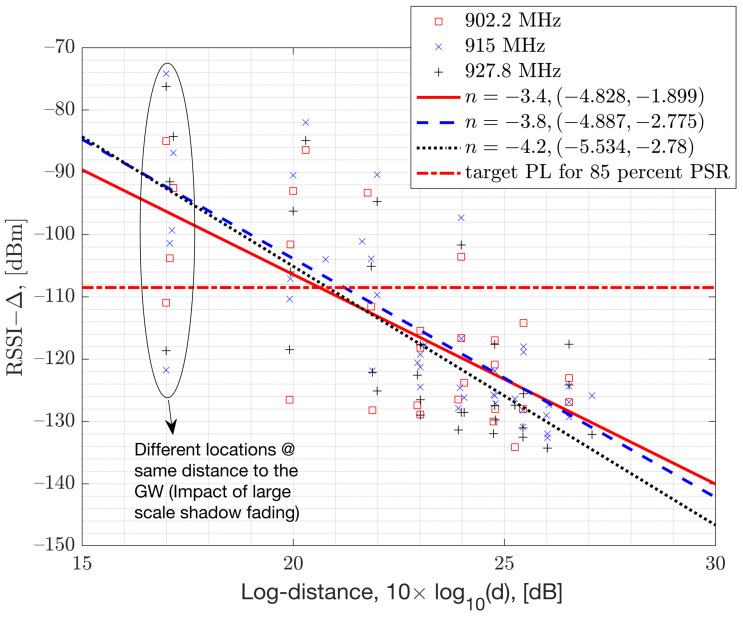
Path loss measurements plotted against the logarithm of the distance from the GW in an urban neighborhood.

To address the variations in the path-loss exponent, a more intricate path-loss model that considers terrain type and the presence of structures such as buildings and tall trees can be devised. This might involve incorporating a geographical map. Instead of opting for such a complex approach, we utilize a statistical method to model the mean path-loss exponent. This involves collecting data on the path-loss exponent from different locations and leveraging these data to generate an estimate for the expected path-loss exponent. The mean path-loss exponent, denoted as n, is determined by estimating the average slope of the samples in relation to the logarithmic distances to the GW. This estimation is achieved through Least Squares Linear Regression. The plot of these measured RSSI samples against the log-distance to the GW is depicted in Figure 6. Organizing the samples based on log-distance enables the estimation of the slope of the linear log-distance line, expressed as: $10\times n\times log_{10}\left(\frac{d}{d_{0}}\right).\;$Figure 6 illustrates the estimated mean-path-loss exponent, featured in the legends of each measurement dataset, along with their respective 95% confidence intervals for each channel.

**Figure 7:**
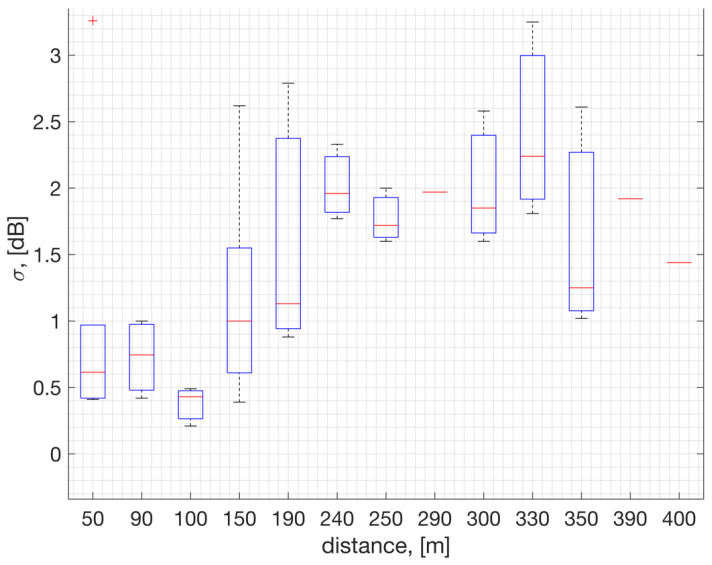
RSSI sample standard deviation across [902, 902.2, 915, 927] MHz channels, analyzed with respect to distance to the GW.

The 95% confidence intervals show that the mean path-loss exponent can vary from a value of n=−2 all the way up to n=−5.5, with an overall mean value of n=−4 averaged across channels. This variation is attributed to differences in terrain, surrounding buildings, and interference levels. In numerical results, we assume a mean path-loss exponent value of n=−4 and log-normal shadow fading with a standard deviation σ=1.4 dB (ref. Figure 7). This simplifies the model. In Figure 6, the term Δ represents the additional path-loss coming from the hardware losses such as RF insertion losses, as the signal travels from the antenna port to the hardware’s chipset port.

**Figure 8:**
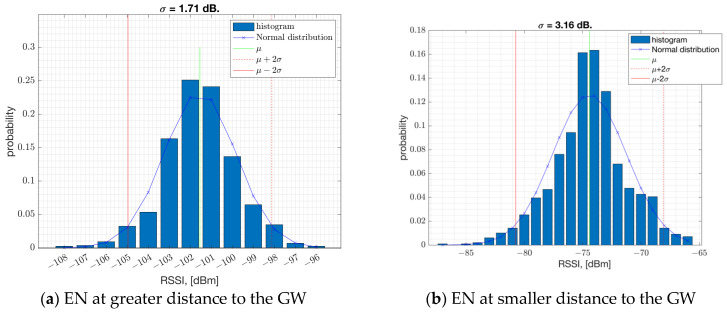
The histogram of the RSSI samples at two different locations.

The field measurements taken in an urban area such as Westchester, LA, show that the shadow fading standard deviation is estimated to be σ = 1.4 dB averaged across locations (ref. Figure 7). This estimate is based on measurements within 400 m to the GW, where the RSSI is represented in full range to cover the PSR of 100% down to 0%. In Figure 7, on each box, the central red line indicates the median, and the bottom and top edges of the box indicate the 25th and 75th percentiles of the measurements, respectively. The whiskers extend to the most extreme data points not considered as outliers, and the outliers are plotted individually using the ‘+’ symbol. Figure 8 illustrates the probability mass function of the RSSI samples collected at two distinct positions (depicted in Figure 8a,b). In the plot, the terms μ and σ represent the mean and standard deviation of the RSSI samples, respectively. The results suggest that both the average RSSI value and its standard deviation are impacted by factors such as location, environment, and proximity to the GW.

## 3. PHY Model with GFSK for Low Bit-Rate IoT Applications

The principles of GFSK modulation have been analyzed in detail in the literature, along with detailed analytical models [1,19,30]. In this section, GFSK modulation is introduced for IoT applications along with the selected modulator parameters. A PHY model with a 50 kbps bitrate is assumed, which is suitable for indoor and outdoor IoT applications [1,16]. First, the sensitivity performance is presented, and the simulation results are compared to lab measurements. Second, the co-channel interference measurement method is presented along with conducted lab measurements. Both the co-channel interference and the sensitivity models provide the building blocks for coverage area and network capacity analyses. Such a coverage area analysis will be presented in Section 6. The additional link budget losses arising from interference from other networks are accounted for in the path-loss model.

(A) GFSK Parameters selection

In this section, the crucial PHY parameters are presented for GFSK modulation for narrow-band, low-bitrate IoT applications. For GFSK modulation, the modulation index, h, and the frequency deviation, $f_{d}$, are defined as follows: $f_{d}=h\times \frac{1}{2}\times \frac{1}{T}\times \left(2^{m}-1\right)$ [Hz]. In this paper, the term m is set to 1 bits/symbol for 2-GFSK, and *0* < h ≤ *1*. The modulation index determines the maximum phase shift between two adjacent symbols in time, given by ϕ=±π×h. When the modulation index is set to maximum value of 1, the modulator runs with a maximum frequency deviation of ±25 kHz with a 50 kbps PHY rate. Adjusting the modulation index to a lower value, such as h=0.5 instead of 1, results in a decreased frequency deviation in the modulated bits. While this reduction can be disadvantageous in the presence of RF impairments like carrier frequency offset (CFO), especially in narrow bandwidth scenarios, a smaller modulation index—such as 0.5—can improve the Adjacent Channel Leakage Ratio (ACLR) performance. This improvement is crucial for applications with higher bit rates in the realm of Internet of Things (IoT), such as those utilizing Bluetooth Low Energy (BLE) with bandwidths up to 2 MHz [19].

**Figure 9:**
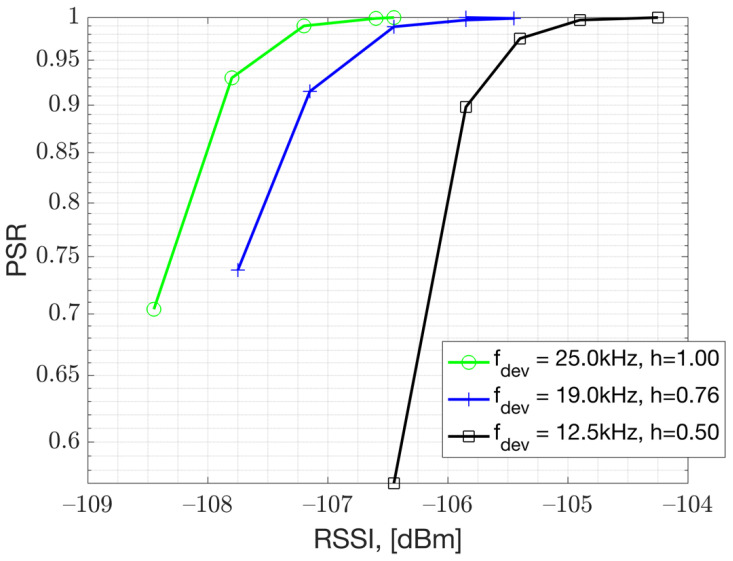
PSR versus RSSI measurements in relation to modulation index, h, using GFSK 50 kbps with BT = 0.5.

Based on the conducted PSR versus RSS measurements presented in Figure 9, it is recommended to keep the modulation index at h=1 for GFSK 50 kbps implementation. The PSR results, as shown in Figure 9, indicate that h=1 achieves a better performance than h=0.76 and h=0.5. This makes sense, as a larger frequency deviation results in more separation among the soft FSK bits which are under a White Gaussian Noise (AWGN). This is especially crucial for very low bit-rate IoT applications that use very small frequency deviations. These results were obtained using the Texas Instruments Evaluation Board (TI-EVB), supporting GFSK 50 kbps modulation. The testing involved a setup where the Rx signal power input to the EVB was varied. Essentially, this result presents the performance under thermal noise and corresponds to AWGN performance.

**Figure 10:**
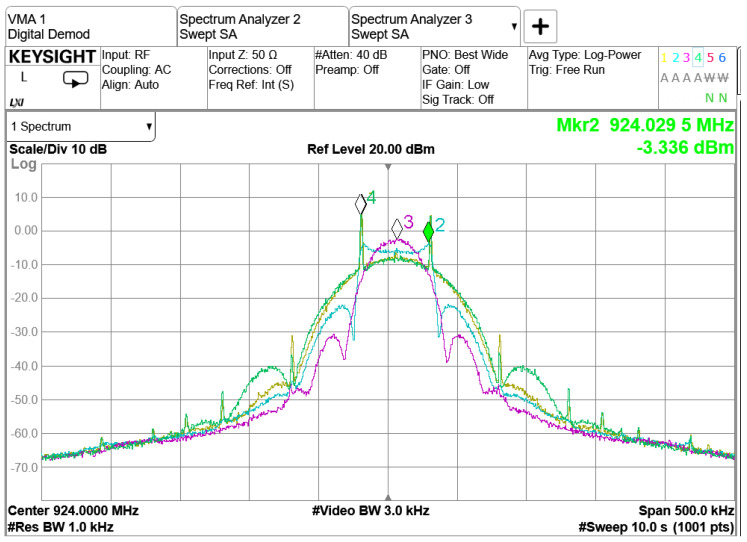
Power Spectral Density measured in relation to the modulation index and pulse shaping filter coefficient for GFSK modulation.

The main factor influencing Rx sensitivity is the modulation index used for GFSK modulation (ref Figure 9). The measurements presented in Figure 9 show that a larger modulation index improves the sensitivity performance for low-bandwidth GFSK modulation. The channel spacing is set to 200 kHz, which is significantly larger than the modulation bandwidth. This allows a larger modulation index to be feasible in terms of ACLR performance. The Gaussian pulse shaper assists with the larger modulation index by limiting the power spectrum density (PSD) (ref. Figure 10). The PSD measurement indicates that the transmitter’s Gaussian pulse shaper Bandwidth-Time product (BT) parameter should be set to BT = 0.5 to enhance the ACLR [1]. This analysis is presented in Figure 10, which is obtained from measurements with a TI-EVB. The traces are measured with the settings presented in Table 1. For instance, when the modulation index is set to 1, the green and yellow traces demonstrate that the second ACLR can be improved by 5 dB when the pulse shaper filter BT is tightened from 1 to 0.5. The accompanying sensitivity measurements indicate that there is no significant difference in Rx sensitivity between a BT value of 1 and 0.5.

(B) Sensitivity Performance

**Figure 11:**
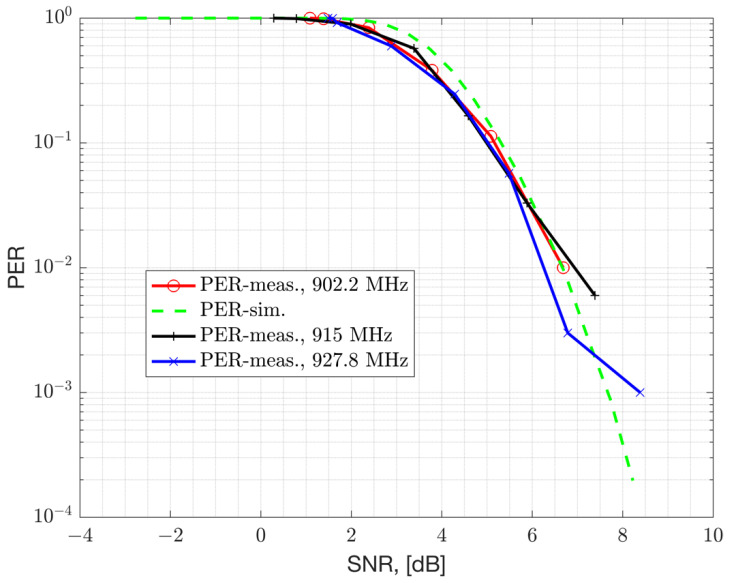
PER measurements versus SNR for GFSK 50 kbps, considering a 64-byte payload, 9 dB Noise Figure, and Rx bandwidth of 155.4 kHz.

**Figure 12:**
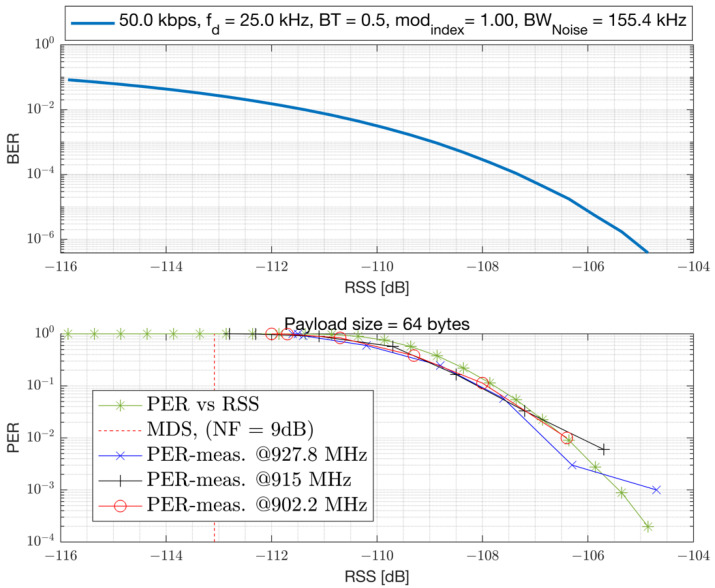
PER and BER in relation to RSS for GFSK 50 kbps, considering a 64-byte payload, 9 dB Noise Figure, and Rx bandwidth of 155.4 kHz.

In this section, the sensitivity performance of the receiver is presented with GFSK 50 kbps modulation. The RSS and the Signal to Noise Ratio (SNR, in dB) are related as follows:

(5) $$RSS=-174[dBm/Hz]+10\times log_{10}\left(BW\right)+NF+SNR,\left[dBm\right]$$

where the term NF represents the Noise Figure (NF) of the device for thermal noise and other noise sources. The chipset under characterization is a TI-EVB and has a 9 dB NF. The PER versus the Signal to Noise Ratio (SNR) and RSS is presented in Figure 11 and Figure 12, respectively. Both the simulation results assuming AWGN channel and conducted lab measurements are presented. The results are obtained with an un-coded baseband, which does not employ Forward Error Correction (FEC). FEC can improve sensitivity at the cost of reduced bit-rate. The Rx filter bandwidth of the device is set to 155.4 kHz for the 50 kbps GFSK mode with the recommended modulation index of 1 and a Gaussian filter coefficient of BT = 0.5. The Rx filter bandwidth is set to a value larger than the modulation bandwidth (50 kHz) to accommodate CFO impairment that can vary across process, voltage, temperature, and frequency (PVTF). However, a larger Rx filter bandwidth passes more thermal noise and causes a degradation in Rx sensitivity, but simplifies the hardware. A modulation index of 1 is selected for such a narrowband modulation to improve robustness to RF impairments such as CFO. The receiver sensitivity of the device can be related to the RSS level required to achieve a bit error rate of $BER\leq 10^{-4}$. This corresponds to an un-coded PER of 1% with a 64-bytes payload. From the results, it can be seen that this device can achieve an un-coded sensitivity of −107 dBm (at 155.4 kHz Rx bandwidth) and requires an SNR of 6 dB with 9 dB NF. The simulation results presented in the AWGN channel are compared with the conducted laboratory measurements, demonstrating a strong agreement.

(C) The Co-channel Interference Performance

**Figure 13:**
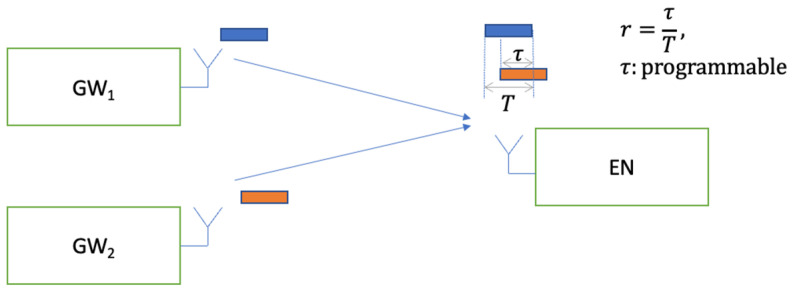
Setup for co-channel interference measurement.

In this section, the measured performance of the GFSK 50 kbps PSR under co-channel interference is presented. The results are measured with the overlap ratios of *r* = 1 for baselining, with *r* = 0.5, and *r* = 0.25 for comparison. The system setup presented in Figure 13 is used for the conducted measurements, where there is one interfering user for link-level system characterization. The term τ represents the duration of the overlapping section of the interferer over the desired packet, where r=τ/T represents the overlap ratio presented in Equation (1). The measurements are conducted for different values of τ and, hence, different values of r.

**Figure 14:**
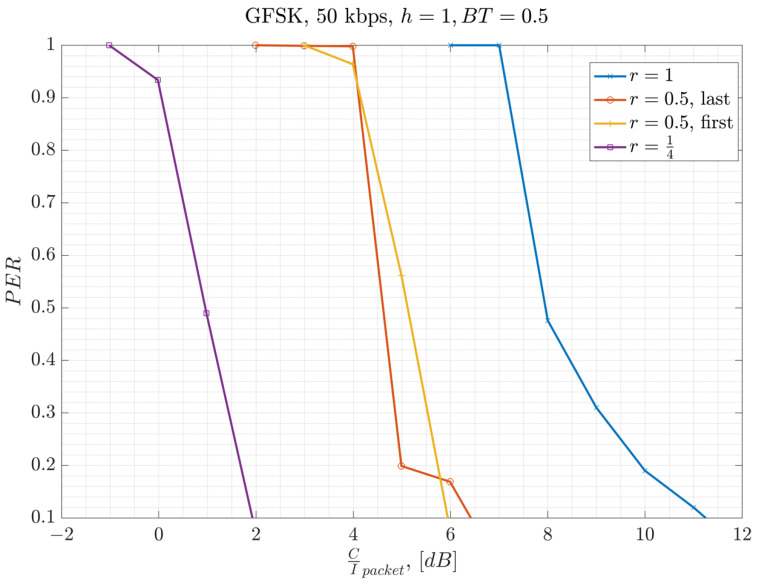
Experimental measurement of PER in relation to Carrier-to-Interference Ratio for GFSK-50 kbps.

The conducted lab measurements with this test setup are presented in Figure 14. In the lab, GW_1_ and GW_2_ represent a signal generator playing a GFSK waveform created by SX-1262 EVB [31]. The waveform is created by using a modulation index of h=1, BT=0.5, and assuming a 50 kbps bit rate (ref. Table 1). The waveforms are scaled relative to each other to achieve the desired C/I level at full overlap. The second waveform from the signal generator is delayed by a time equal to τ seconds relative to the first signal. Then, both waveforms are added to each other after the scaling and delaying of one of them. The resulting waveform is then input into the EN’s receiver, using another SX-1262 EVB. The measurement results are presented in Figure 14. On the *x*-axis, ${\frac{C}{I}}_{packet}$ is calculated based on the partial interfering overlap duration τ and packet duration T by using Equation (1). The results illustrate that Equation (1) represents the performance well, where smaller overlap ratios help to improve the performance. In other words, the required C/I threshold for successful packet reception reduces by as much as $10\times log_{10}(r)$, dB. The measurements show that a worst case C/I threshold of at least $\gamma_{th}=7$ dB is required when there is a full overlap, where r=1.

## 4. The Relationship between the Beacon Collision Probability and Beacon Error Rate

The loss of a beacon causes an increased latency to resynchronize and might result in the falling out of the network; hence, beacon collision probability is investigated in detail in this section. Each beacon must be transmitted periodically; hence, GWs do not employ the LBT mechanism, which does not help in collision avoidance.

Assuming a binomial distribution for the devices that can collide with each other at a given instant in time, we can write the collision probability as follows. Let *N* represent the number of devices that can collide with each other. It can, for example, represent the number of ENs in the neighbor WAN performing UL. Or, *N* represents the number of GWs in the area that are beaconing and can potentially collide with another beacon from another GW. Such beacon-to-beacon collision is referred to as DL collision as well, since beacons are broadcast in the DL direction. Let $p_{c}$ represent the collision probability between individual links. The probability of *x* devices colliding out of *N* devices can be calculated as follows, where x≤N,x∈{1,2,…,N}:

(6) fxN,pc=Nxpcx1−pcN−x

Here, x represents the number of collisions in *N* trials of a Bernoulli process with an individual probability of collision given by $p_{c}$. The term $f\left(x|N,p_{c}\right)$ represents the Probability Mass Function (PMF) of the number of collisions, x.

Then, the total probability of collision summed across all possible values of *x ≤ N* can be calculated as:

(7) FxN,pc=∑x=0NNxpcx1−pcN−x

The term $F\left(x|N,p_{c}\right)$ represents the Cumulative Distribution Function (CDF) of the number of collisions. Here, it is assumed that any transmission on a given channel is independent of the other transmissions.

The mean number of collisions can be calculated as:

(8) $$\mu =N\times p_{c}.$$

A collision is assumed to occur if any packet collides for a duration greater than zero. With this assumption, the individual collision probability per link, denoted by $p_{c}$, can be calculated as follows [32]:

(9) $$p_{c,DL}=\frac{\left(T_{beacon}+T_{beacon}\right)}{T_{beacon\;interval}\times N_{ch}}.$$

The terms $p_{c,UL}$ and $p_{c,DL}$ represent the individual collision probabilities on a beacon from the UL links and the DL links, respectively. The term $p_{c,UL}$ represents the probability of collision between a beacon and an UL packet, while $p_{c,DL}$ represents the collision probability between two individual beacons from two different GWs. The primary GW’s beacon is assumed to occupy $T_{beacon}$ seconds, sent once every $T_{beacon\;interval}$ across $N_{ch}\geq 1$ channels. The term $N_{pkt}$ represents the number of UL packets within each beacon interval, and $T_{pkt}$ represents the duration of each UL packet from each neighbor EN. The number of channels where frequency hopping is exercised is represented by the term $N_{ch}$. A large number of channels or a longer beacon interval decreases the likelihood of collisions. Similarly, a larger number of packets per beacon interval increases the likelihood of collisions in the UL.

**Figure 15:**
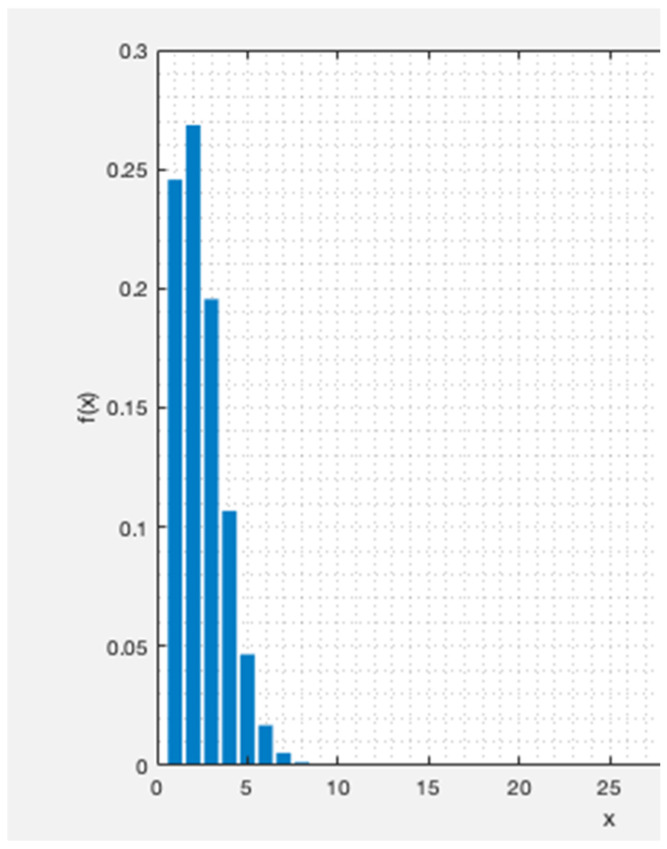
The probability of x devices colliding out of a total N=5000 devices.

For instance, the PMF with *N* = 5000 GWs, denoted as $f\left(x|N=5000,\;p_{c}\right),$ is illustrated versus *x* in Figure 15 for the DL. This plot depicts the distribution of beacon collision probability. The parameters considered for this result are $T_{beacon}$ = 152 ms, $N_{ch}=69$, and $T_{beacon\;interval}=10.08\;s$. With these assumptions, the mean number of collisions is $\mu =N\times p_{c,DL}=1.9$.

The PMF plot in Figure 15 above serves as an illustrative example, revealing that, with N=5000 GWs, the likelihood of x=1 and x=2 devices colliding is around 0.25 and 0.27, respectively. In contrast, the probability of more than 10 devices colliding simultaneously is 0, as determined by the PMF described in Equation (6).

Packet collisions on a beacon happen with the probabilities outlined in Equations (6)–(9). However, not all collisions result in a beacon loss. This is because, when $\frac{C}{I}>\gamma_{th}$, the beacon is successfully received by the EN despite a collision (ref. Figure 5, Section 2-B and Section 3-C). Hence, the overall performance depends not only on the distance to a GW, but also on the interference observed by the ENs. Closer ENs to a GW experience a better *RSS,* increasing their chance of having $\frac{C}{I}>\gamma_{th}$. The expected values of $\frac{C}{I}$ versus the network size is presented in —Section 6).

For a beacon to be lost, the following conditions must be met:

1. The signal must be below the sensitivity level; otherwise,
2. A collision must occur,
3. The C/I on the packet, calculated with the overlap ratio, needs to be worse than a threshold. (The thresholds and the co-channel interference model is presented in Section 3-C)

With this introduction, the beacon loss rate can be approximated as follows:

(10) $$\begin{array}{ll} P_{b,\;err}\left(D\right) & \approx \;\frac{1}{N_{en}}\sum_{k=1}^{N_{en}}\int_{y=\frac{-D}{2}}^{y=\frac{D}{2}}\int_{x=\frac{-D}{2}}^{x=\frac{D}{2}}p_{r}\left(RSS{\left(x,y\right)}_{k}<\beta \right)dxdy+P_{c}\left(N\right)\\ & \times \;\frac{1}{N_{en}}\sum_{k=1}^{N_{en}}\int_{y=\frac{-D}{2}}^{y=\frac{D}{2}}\int_{x=\frac{-D}{2}}^{x=\frac{D}{2}}{p_{r}\left(RSS{\left(x,y\right)}_{k}\geq \beta \right)p}_{r}\left(\frac{C}{I}{\left(x,y\right)}_{k}<\gamma_{th}\right)dxdy. \end{array}$$

The terms x and y represent the location of each EN relative to the GW to which it is connected. The term *D* represents the size of the area border in meters where the beacon error rate calculation is made. The term $p_{r}\left(RSS{\left(x,y\right)}_{k}>\beta \right)$ represents the probability at which the RSS (in dBm) is greater than the sensitivity, represented by the term β (in dBm). The probabilistic sum of all possible collisions for a total of *N* gateways is represented by the term $P_{c}\left(N\right)=F\left(x|N,p_{c}\right)$, given in Equation (7). The term $p_{r}$ represents the probability of its argument. For example, $p_{r}\left(\frac{C}{I}{\left(x,y\right)}_{k}<\gamma_{th}\right)$ represents the probability at which the carrier to interference ratio ($\frac{C}{I}$) at location ${\left(x,y\right)}_{k}$ observed at the EN $k\;(k\in \{1,2,\ldots ,N_{en}\}),$ is less than a threshold $\gamma_{th}$ dB. Our PHY layer measurements show this threshold to be $\gamma_{th}\approx$ 7 dB (ref. Section 3). The closed-form expression for calculating C/I while considering the overlap ratio is given in Equation (1).

## 5. Simulator Architecture

**Figure 16:**
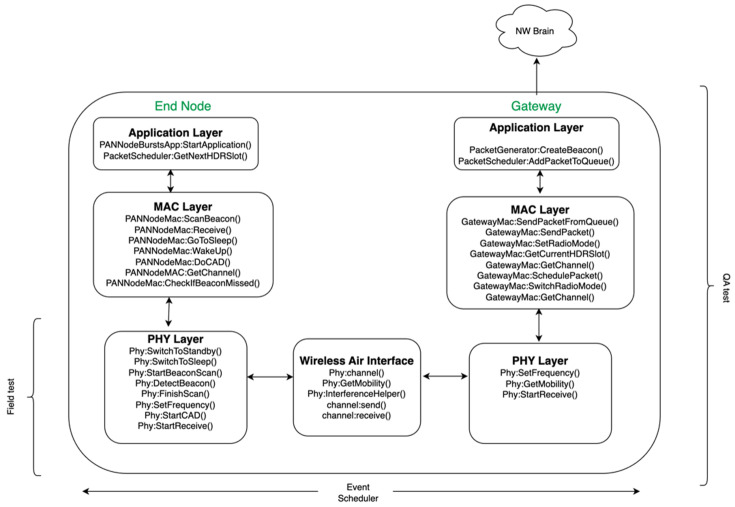
Diagram illustrating the NS-3 Simulator Architecture, spanning from the application layer to the physical (PHY) layer.

In this section, the simulator architecture is introduced. The simulator is developed to generate the numerical results presented in Section 6. The Network Simulator-3 (NS-3) is utilized based on the NS-3.33 version. NS-3 is a discrete-event-based network simulator that operates based on a series of events created by a specific network topology [33]. New classes and methods are implemented to create the WAN IoT simulator along with the specific PHY model, as presented in Section 3. Modifications are applied to the simulator to incorporate the PHY and MAC layer models presented in Section 2. The classes created for each layer of the protocol stack are depicted in Figure 16. The application layer manages the events for packet generation and schedules them down to the MAC layer. The MAC layer at the GW is responsible for beacon creation, frequency hopping, and switching among various radio modes. The MAC layer at the EN is responsible for scanning for beacons, receiving a beacon, or a data packet coming from the GW in the DL, and setting the channel, etc. The event scheduler manages the MAC and PHY layers, i.e., it manages switching among various radio modes such as sleep, wake-up, standby, CAD, active RX, and active TX, etc. The PHY layers at the EN and GW are interconnected via the wireless channel, i.e., the wireless air interface. The wireless air interface computes the distances between the GWs and ENs and determines the corresponding RSSs. The C/I ratio per link at a given time is calculated using the interference helper function. This function calculates the air times per packet and determines all the overlapping collision events over which the C/I value is determined as described in Section 2. The PSR is determined based on the C/I and RSS levels experienced by each packet. The PSR is calculated as the ratio of the total number of packets successfully received versus the total number of packets sent.

This simulator has been validated with the Quality Assurance (QA) test setup, where the PER obtained with the simulator is compared to that of the QA test result. The PHY model is developed based on the measurements obtained from the field, as presented in the earlier section on the PHY model with GFSK.

## 6. Numerical Results

**Figure 17:**
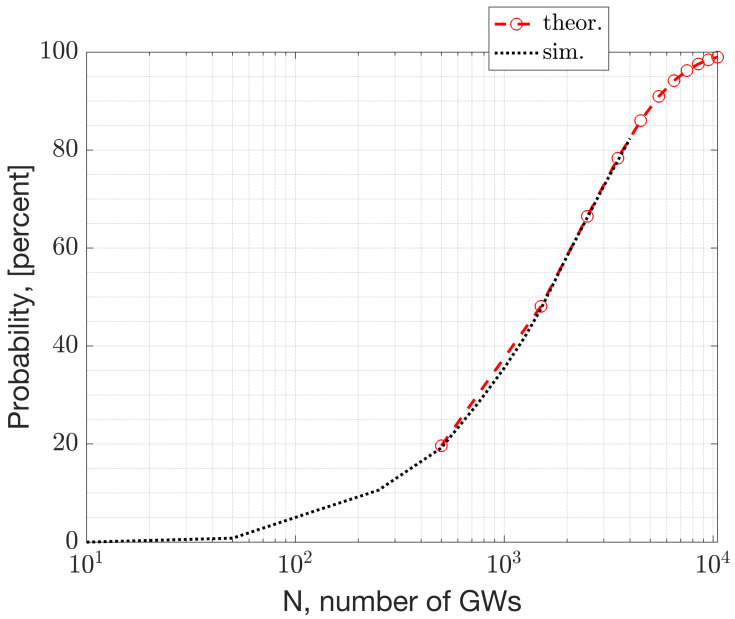
The beacon collision probability in relation to the number of Gateways, *N*.

**Figure 18:**
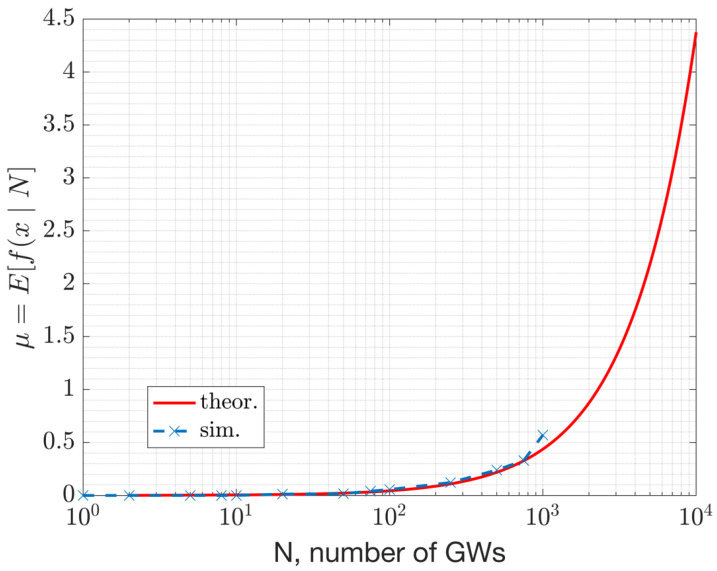
Expected number of collisions in relation to the number of Gateways.

This section presents numerical results derived from a simulation study using NS-3. Figure 17 and Figure 18 depict the beacon collision probability and the expected number of devices under collision, respectively, versus the network size, where the network size refers to the number of GWs and ENs. The collision probability represents the scenario where two or more packets with an infinitesimal overlap collide. Consequently, the collision probability serves as an upper bound for the packet error rates. The packet error rate depends on the product of the collision probability and the probability of having a C/I that is unfavorable for a successful reception. Further analysis of the PER’s dependency on the sensitivity is given in Equation (10).

The system parameters used to derive the performance evaluation results are described below. We assume the presence of 160 sub-slots for UL contention windows, a beacon duration of 152 ms, and a beacon interval of 10.08 s. There are 69 channels available for FHSS. While a higher number of channels has the potential to improve performance, a careful balance is required to weigh the benefits against the increased complexity and the power consumption demands, particularly for battery-operated ENs.

**Figure 19:**
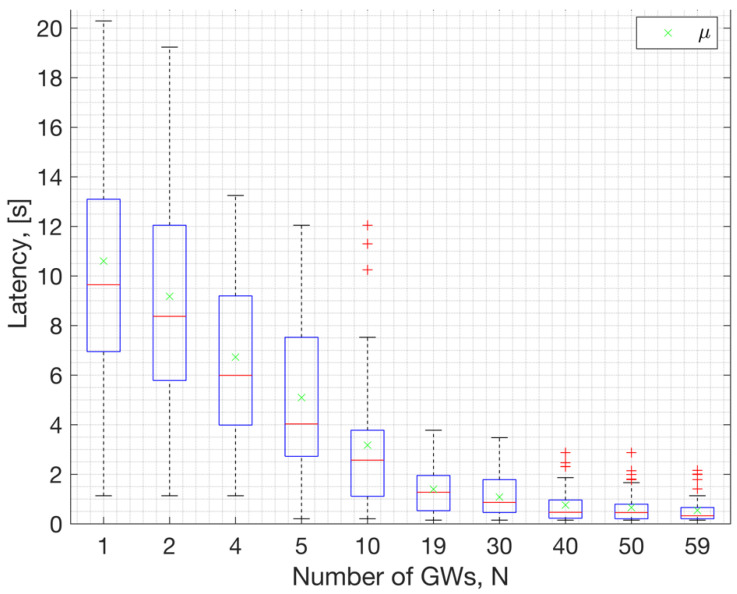
Latency in beacon acquisition relative to the number of Gateways with 50 ENs.

Statistics for beacon acquisition latency, derived from Monte Carlo simulations using the NS-3 model, are depicted in Figure 19. On each box in the figure, the central red line indicates the median, and the bottom and top edges of the box indicate the 25th and 75th percentiles of the samples obtained from the simulation results, respectively. The samples, i.e., the data points marked with + symbols in this plot represent outliers within the dataset. Outliers are observations that deviate significantly from the overall pattern of the data. The green ‘x‘ symbols on the data points represent the dataset’s mean values obtained through simulations for a specified number of GWs. Opting for a longer beacon interval, such as 10.08 s, results in a mean beacon acquisition latency of approximately μ= 10.6 s when 50 ENs are attempting to discover a single GW. Conversely, beacon acquisition latency diminishes exponentially with an increasing number of GWs available to serve the WAN in the neighborhood. Thus, choosing an extended beacon interval, along with a frequency-hopping system featuring multiple channels, proves to be a favorable option for a narrow band WAN, especially in scenarios where multiple GWs can serve the ENs.

**Figure 20:**
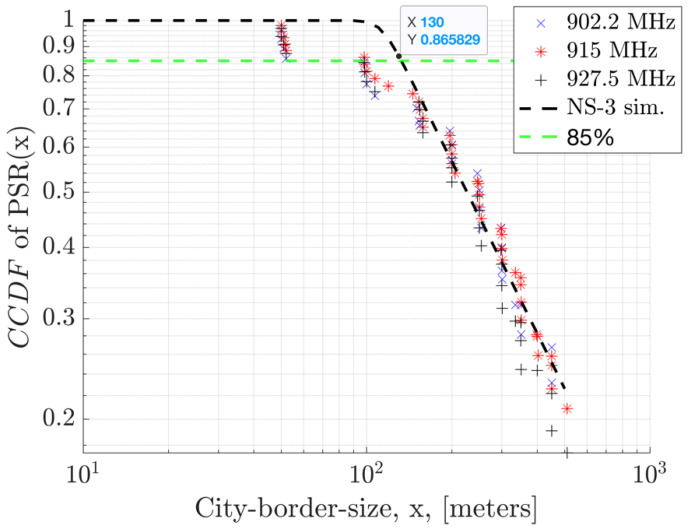
CCDF of PSR in relation to the distance from the GW in urban terrain, considering 1 GW, 1 EN, and a 64-byte payload.

In Figure 20, the Complimentary Cumulative Distribution (CCDF) illustrates field measurements for PSR in relation to the distance from the nearest GW, using 50 kbps GFSK modulation. Within this graph, the 85th percentile of the PSR, denoted as TP-85, is highlighted. Observations from various locations indicate that a coverage range of approximately 130 m can be attained with PSR values of≥0.85. In other words, the TP-85 distance covered is 130 m with PSR values greater than or equal to 85%. These measurements were conducted in Westchester, Los Angeles, an urban area. Multiple measurements were taken at various locations equidistant from the GW to showcase the statistics and impact of interference in a real-life scenario. The results are compared to the NS-3 simulation outcomes, where a fixed additional loss is presumed for the environmental interference. The NS3 model sets the path loss exponent to the mean value predicted by experimental results, while in the field, variations in the terrain and buildings can lead to differences in the measurement outcomes. The field measurements show that a CCDF equal to 1 (100% PSR) is not commonly observed due to non-zero interference, even when in the vicinity of the GW.

**Figure 21:**
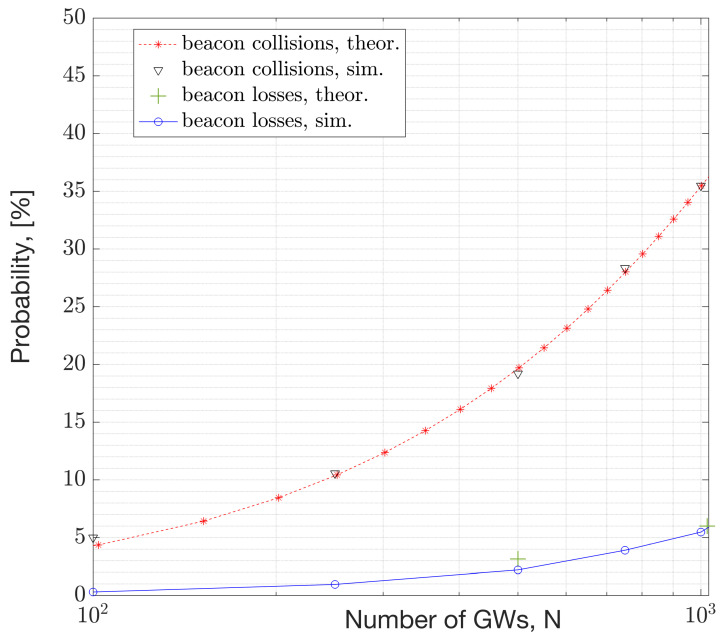
Relationship between beacon collision rate and beacon error rate versus number of gateways.

Figure 21 depicts the simulation results for both the beacon collision probability and beacon loss rate. The beacon loss rate is simulated with the path-loss model presented in the System Model section (Section 2.C). A collision is identified if there is a non-zero temporal overlap between the packets. Not every collision results in packet loss. The relationship between the collision rate and packet loss rate is presented in Equation (10) in Section 4. The beacon loss rate simulation results are presented in Figure 21. They are obtained by assuming a fixed area size while increasing the GW density. The simulation result aligns with the theoretical outcome from Equation (10), illustrated with the green ‘+’ markers. Calculations are derived from the distribution of C/I samples. The simulation results demonstrate that 1000 GWs can coexist (at ~$\frac{35\times 35\;\mathrm{GWs}}{{km}^{2}}$ density) with a 5% beacon loss rate, equivalent to a beacon success rate of 95%.

## 7. Conclusions

This paper demonstrates that DL-beacon performance under interference depends on several factors, such as the PHY bit-rate available, the protocol parameters (including the number of channels, the beacon duration and its periodicity, and the number of devices such as the number of GWs and ENs), and the environment (urban, sub-urban, or rural areas, which provide different path-loss environment and different levels of interference from other networks).

This study demonstrates that, with large beacon intervals and an ample number of channels for frequency hopping, a considerable number of GWs can coexist in a WAN IoT network. For instance, a beacon interval of 10.08 s was analyzed along with 69 channels and a 152 ms beacon duration. Under such system settings, the expected number of colliding GWs (or beacons) in the DL is 1 out of 2000 GWs (Figure 18). This result corresponds to the performance of the MAC layer. According to the results with the path-loss model, 1000 GWs can coexist (at ~1225 GWs/km^2^ density) with an average beacon success rate of 95% (ref. Figure 21). This capacity result corresponds to the combined performance of the MAC and PHY layers. The performance relies on the protocol settings and HW settings. Due to the large beacon interval, the results indicate that the mean beacon acquisition latency is 10.5 s with only one GW. This latency significantly decreases when multiple GWs are within the reach of a given EN (Figure 19). The availability of GW diversity enhances the coverage area and reduces beacon acquisition latency.

Future works as next steps to this study include, but are not limited to, the following. 1. Conducting an analysis of the capacity of a multi-rate GW capable of supporting different types of ENs, ranging from those that support higher bitrates to those supporting lower bitrates. 2. Assessing the performance versus beacon interval to identify the optimal balance between latency and network capacity under interference. 3. Proposing a next generation system design where GWs are coordinated in the cloud and employ a frequency reuse mechanism to minimize the interference impact of neighboring GWs. 4. Providing a performance evaluation of UL and DL data links with LBT and determining the maximum number of packets per beacon interval that can be supported in the network. 5. Conducting a performance evaluation for urban dense and rural environments.

## Figures and Tables

**Table 1:** Modulation parameters tested in the laboratory using TI-EVB for GFSK 50 kbps IoT modulation.

	Pulse Shaper BT Parameter	Modulation Index, h	Frequency Deviation, kHz	Bitrate
Trace-1 (Yellow trace)	BT = 0.5	1	±25	50 kbps
Trace-2 (Blue trace)		0.76	±19	
Trace-3 (Magenta trace)		0.5	±12.5	
Trace-4 (Green trace)	BT = 1	1	±25	

## Data Availability

Data are contained within the article.
